# Longitudinal evaluation of Supportive care Prioritization, Assessment and Recommendations for Kids (SPARK), a symptom screening and management application

**DOI:** 10.1186/s12885-019-5662-9

**Published:** 2019-05-16

**Authors:** Emily Vettese, Sadie Cook, Dilip Soman, Susan Kuczynski, Brenda Spiegler, Hailey Davis, Nathan Duong, Tal Schechter, L. Lee Dupuis, Lillian Sung

**Affiliations:** 1 Program in Child Health Evaluative Sciences, The Hospital for Sick Children, Peter Gilgan Centre for Research and Learning, 686 Bay Street, Toronto, ON M5G 0A4 Canada; 2 Rotman School of Management, University of Toronto, 105 St. George Street, Toronto, ON M5S 3E6 Canada; 3 Ontario Parents Advocating for Children with Cancer (OPACC), 99 Citation Drive, Toronto, ON M2K 1S9 Canada; 4 Department of Psychology, The Hospital for Sick Children, 555 University Avenue, Toronto, ON M5G 1X8 Canada; 5 Division of Haematology/Oncology, The Hospital for Sick Children, 555 University Avenue, Toronto, ON M5G 1X8 Canada; 6 Department of Pharmacy, The Hospital for Sick Children, 555 University Avenue, Toronto, ON M5G 1X8 Canada

**Keywords:** Pediatric cancer, Symptom screening, Supportive care, Self-report, Oncology, Patient reported outcomes

## Abstract

**Background:**

Supportive care Prioritization, Assessment and Recommendations for Kids (SPARK) is a web application focused on improving symptom control. It enables pediatric cancer and hematopoietic stem cell transplant (HSCT) patients to self-report and track symptoms, and allows healthcare professionals to access guidelines for symptom management. Objective was to determine the feasibility of longitudinal collection of symptom data.

**Methods:**

In this longitudinal, single-armed feasibility study, respondents were children 8–18 years of age with cancer or pediatric HSCT recipients. Participants completed symptom reporting daily for 5 days. Cognitive interviews were conducted on day 5. Quantitative evaluation included SPARK ease of use and understandability of SPARK reports. Qualitative feedback on facilitators and barriers to daily symptom screening was solicited. Feasibility was defined as ≥75% of participants completing symptom screening on at least 60% of on-study days during the five-day study.

**Results:**

Among the 30 children enrolled, the median number of days SSPedi was completed at least once was 5 (range 3 to 5). Overall, 28/29 (96.6%) thought completing symptom screening using SPARK was easy or very easy. All participants understood SPARK symptom reports. Severe symptoms was the most common barrier to daily reporting while an alarm reminder system was the most commonly identified facilitator.

**Conclusions:**

Daily completion of symptom screening using SPARK over 5 days was feasible in children aged 8 to 18 years with cancer and pediatric HSCT recipients. SPARK is now appropriate for use in randomized trials to evaluate the effect of symptom screening and symptom feedback.

## Background

Children with cancer and pediatric hematopoietic stem cell transplantation (HSCT) recipients frequently experience severely bothersome symptoms [1]. Among children admitted to hospital for at least 4 days, 99% reported at least one bothersome symptom and 60% reported at least one severely bothersome symptom [2]. In order to improve symptom control, we need to enable children to self-report and track symptoms, improve communication of these symptoms to healthcare professionals and facilitate access to clinical practice guidelines for symptom control. Two previous systematic reviews have highlighted the limited number of studies addressing symptom screening in pediatric oncology, and described whether data were collected longitudinally and whether studies used electronic data collection [3, 4].

To address the gaps related to symptom self-report and communication, we developed SSPedi (Symptom Screening in Pediatrics Tool) [5], a symptom screening tool developed specifically for children receiving cancer treatments [6, 7]. Building upon SSPedi, we developed SPARK (Supportive care Prioritization, Assessment and Recommendations for Kids), a web application consisting of a symptom-screening and tracking component centered on SSPedi and a supportive care clinical practice guideline component.

We recently described the initial development of the patient-facing portal of SPARK. Iterative refinements were based on cognitive interviews with 90 children between 8 and 18 years of age receiving cancer treatments and pediatric HSCT recipients [8]. Evaluation was cross-sectional; it focused on design, navigation, usability and likability of both the web application pages and SPARK symptom reports. In the final cohort of 10 children, all understood how to navigate the website, access reports and interpret reports, all found SPARK easy to use and 90% found SPARK reports easy to understand. While these results were promising, we next needed to develop and test aspects of SPARK that promoted repeated conduct of symptom screening longitudinally, including password creation and a symptom screening reminder system. We also wanted to determine whether a future randomized trial of longitudinal symptom screening would be feasible.

The objectives were to determine the feasibility of a future randomized trial of longitudinal symptom screening by describing the proportion of children who completed symptom screening on at least 60% of on-study days during a five-day longitudinal study and to refine SPARK to improve longitudinal utilization from the perspective of children receiving cancer treatments or undergoing HSCT.

## Methods

### Design

This was a longitudinal, single armed feasibility study conducted at The Hospital for Sick Children (SickKids) in Toronto, Canada. The Research Ethics Board at SickKids approved this study; children or guardians provided informed consent or assent as appropriate.

### Eligibility

We included English-speaking children with cancer and pediatric HSCT recipients who were 8–18 years of age at enrollment and who were expected to be in clinic or admitted to the hospital for five consecutive days. Participants could have been actively receiving cancer treatments or could have completed cancer therapies. Exclusion criteria were illness severity, cognitive disability or visual impairment that precluded utilization of SPARK according to the primary healthcare team.

### SSPedi and SPARK

SSPedi is a symptom screening tool that asks about the degree of bother yesterday or today related to the following 15 symptoms: disappointed or sad, scared or worried, cranky or angry, problems thinking, body or face changes, tiredness, mouth sores, headache, other pain, tingling or numbness, throwing up, hunger changes, taste changes, constipation and diarrhea [6]. It is reliable, valid and responsive to change in English-speaking children 8–18 years of age [1].

The overall goal of SPARK is to bring together identification of symptoms with provision of interventions to prevent and treat detected symptoms. Currently, the guidelines that exist within SPARK are those endorsed by the Children’s Oncology Group and are formatted for healthcare professionals. However, an important current initiative is to translate the professional-directed recommendation summaries for both families and children themselves.

### Design

Potential participants were approached in the outpatient clinic or inpatient ward by a member of the research team. Consenting participants completed symptom screening at least once daily on a study-supplied iPad, for five consecutive days. Five days was chosen to allow longitudinal daily evaluation but preserve feasibility as few children are admitted or seen daily for longer than 5 days. For inpatients, an iPad was left with the participant over the 5 days and thus SPARK symptom reports were available to the child at any time. For outpatients, research associates provided the iPad in person daily and reports could be viewed at those encounters.

Participants were asked to create a personalized username and password in order to register to the SPARK website. To protect the participant’s privacy, the username and password could not include any personally identifying information such as their first name or last name. If the participant forgot their username or password, they could click “Forgot my Password” on the SPARK website, refer to a password reminder card that had been provided to them or contact a study team member with the contact information provided on each study iPad. Each participant was shown how to log on, complete SSPedi and access symptom reports.

In order to encourage daily symptom screening over the 5 days, a reminder system was established for each participant using the study iPad’s alarm feature. For inpatients, participants were asked to choose a time before 3:00 pm to set a reminder alarm. The 3:00 pm time was chosen to ensure a member of the research team was still in the hospital in case a problem was encountered and to allow enough time for follow-up if required. Each alarm was set using standardized procedures as follows: the alarm was labelled “Do SSPedi Now”, the snooze feature was turned off, a pleasant alarm tone was chosen and the alarm was set to repeat every day. If the participant did not complete SSPedi by their chosen reminder time, a member of the study team followed up with the participant in person. For outpatients, the daily visit by the research team member served as their reminder to complete SSPedi.

Individual interviews were conducted on day 5 using a semi-structured format with a clinical research associate with expertise in qualitative methods and the think aloud technique of cognitive interviewing. First, the interview focused on general questions. The interviewer asked if the participant viewed any of the reports and whether any of the reports were shared with others including healthcare professionals. Next, we asked about the participant’s experience with SSPedi completion. We asked about potential facilitators and barriers to daily reporting including whether daily completion was too much, too little or about right, and the likelihood of daily completion.

**Fig. 1:**
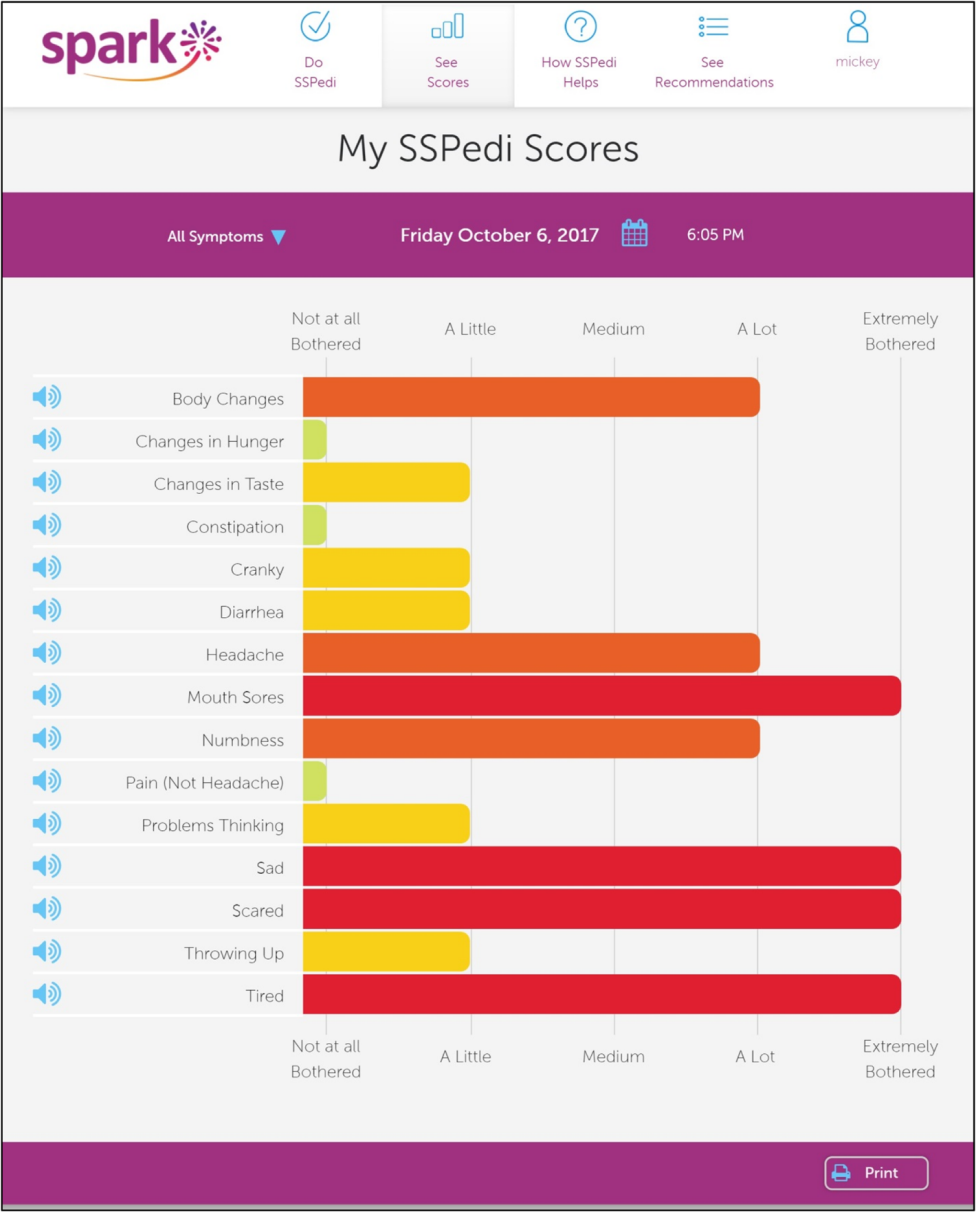
Single administration report. Results after completing each SSPedi administration, showing the degree of bother for each of the 15 symptoms included in the tool

**Fig. 2:**
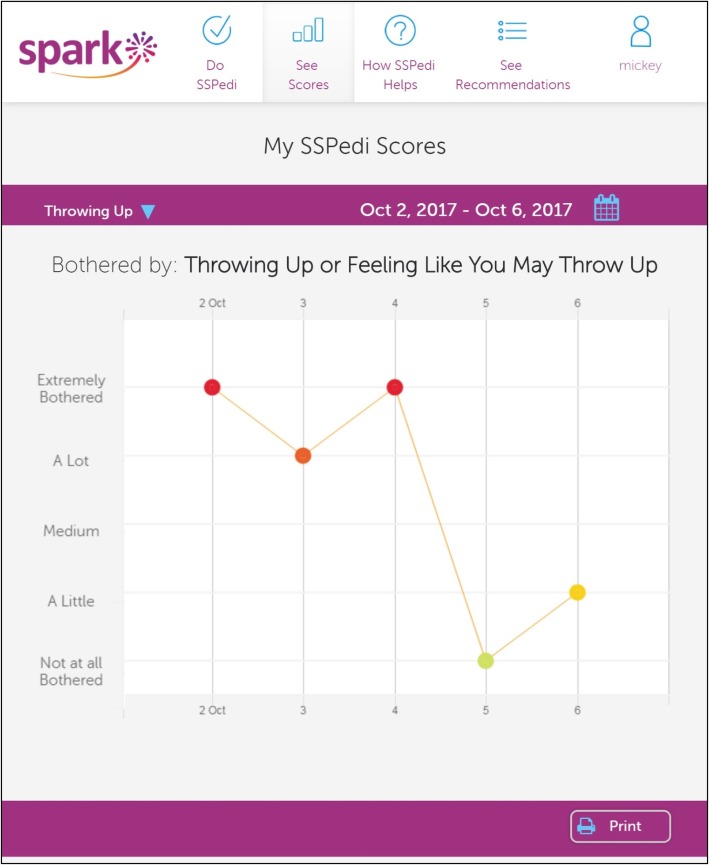
Multiple administration report over a maximum of 5 days in this study. Figure shows the degree of bother for a single symptom included in SSPedi over time. In this example, throwing up or feeling like you might throw up, is shown over a 5 day period

Then, the participant viewed their last SPARK symptom report (single administration report, Fig. 1) and the interviewer asked the respondent to interpret the overall report and a specific symptom. Next, the participant viewed a symptom over time report (multiple administration report over a maximum of 5 days in this study, Fig. 2) and was asked to interpret the report and a specific day’s symptom. Report understanding was evaluated using cognitive interviewing and the think aloud technique. Interpretation was adjudicated independently by two interviewers on a 4-point Likert scale ranging from 1 = “completely incorrect” to 4 = “completely correct”. The two interviewers compared their scores following the interview and if there were discrepancies, they resolved them by consensus. Suggestions for modifications to improve clarity and meaning were solicited. Finally, we asked participants to rate ease of use of SPARK on a 5-point Likert scale ranging from 1 = “very hard” to 5 = “very easy”. They also rated usefulness for children receiving cancer treatment on a 5-point Likert scale ranging from 1=“not useful at all” to 5 = “extremely useful”.

### SPARK modifications

Interview results were summarized and reviewed after every five interviews by research team members to determine whether the interview approach or the script required modification. After 15 and 30 participants were enrolled, a Review Panel met to evaluate the comments and decide whether to modify SPARK. The Panel included two pediatric oncology survivors (ND and HD), one parent advocate (SK), a pediatric psychologist (BS), a behavioral scientist (DS), a pediatric pharmacist (LLD) and a pediatric oncologist (LS). The behavioral scientist was an expert in choice architecture, which is the process of designing choice context to nudge individuals toward making better decisions [9].

### Statistical considerations

We described the proportion of children who completed symptom screening on at least 60% of on-study days, or three of 5 days. Based upon our experience with SSPedi, we conservatively estimated that at least 75% of participants should be able to achieve this level of compliance. We also described the proportion of participants who correctly interpreted SSPedi reports (mostly correct or completely correct). We also described the total SSPedi score, which is the sum of each item’s Likert score for a total score that ranges from 0 to 60 in which higher numbers denote more bothersome symptoms.

We planned to include 30 participants. Assuming that 75% of participants achieved SSPedi completion on at least 60% of days, this sample size provided a two-sided 95% confidence interval with limits of 56–89%; this precision was adequate for our purposes.

## Results

Between November 6, 2017 and March 12, 2018, 53 potential participants were assessed for eligibility; 11 were not eligible due to cognitive disability (*n* = 6), inability to understand English (*n* = 1) and palliation due to progressive disease (*n* = 4). Thus, 42 patients were approached with 12 refusing to participate, resulting in 30 patients enrolled.

Table 1 illustrates the characteristics of the study cohort. The median age at enrollment was 12.1 (range 8.1 to 18.2) years. The median number of days SSPedi was completed at least once was 5 (range 3 to 5), and the median number of times SSPedi was completed in total was 5 (range 3 to 8). Thus, all patients completed symptom screening on at least 3 days, resulting in the proportion of patients achieving our threshold for longitudinal SSPedi completion of 30/30 (100.0%), meeting our feasibility endpoint. Two patients chose to complete symptom screening more than once a day for at least 1 day. The number of children who completed symptom screening all 5 days was 28/30 (93.3%). One inpatient participant completed symptom screening four out of the 5 days due to a 1 day admission to the intensive care unit during the study. One outpatient participant completed symptom screening three out of the 5 days because they did not come in for their scheduled clinic visit on 2 days of the study including day 5. Overall, the mean (± standard deviation) time for SSPedi completion was 1.0 ± 1.5 min and the median total SSPedi score was 10 (range 0 to 36). There were no difficulties encountered with password creation or setting of reminders. Among the 19 inpatients, six required a study team member to remind them to complete SSPedi seven times during 95 symptom screening days. No patient used the password retrieval function of SPARK or contacted the research team to obtain their password after initial creation.

**Table 1:** Demographics of the study cohort (*N* = 30)

Characteristic	n (%)
Male	18 (60.0)
Age in Years	
8–10	8 (26.7)
11–14	11 (36.7)
15–18	11 (36.7)
Diagnosis	
Leukemia or lymphoma	16 (53.3)
Solid tumor	11 (36.7)
Brain tumor	0 (0)
Other^a^	3 (10.0)
Relapse	3 (10.0)
Treatment Group	
Cancer	25 (83.3)
Stem cell transplantation	5 (16.7)
Inpatient	19 (63.3)
Active Treatment	29 (96.7)
Language Spoken at Home	
English	25 (83.3)
Other^b^	5 (16.7)

^a^Other diagnoses were severe aplastic anemia, Wiskott-Aldrich syndrome and severe combined immunodeficiency

^b^Other languages were Spanish, Arabic, Gujarati, French and Tagalog

The interview was conducted with 29 children as one outpatient participant did not attend the scheduled clinic appointment on day 5 of the study. Table 2 illustrates components of the interview focused on SPARK symptom reports. Among the nine participants who shared reports, seven shared it with a family member, one shared it with their physiotherapist and one did not specify with whom the report was shared. All respondents understood the single administration and multiple administration reports. Table 2 also shows results describing experience with longitudinal SSPedi completion. Overall, 28/29 (96.6%) thought completing SSPedi within SPARK was easy or very easy.

**Table 2:** Interview assessments (*N* = 29)

Outcomes	n (%)
SPARK Reports	
Participant viewed SPARK symptom report	23 (79.3)
SPARK symptom report shared with others	9 (31.0)
Correct understanding of SPARK reports assessed in cognitive interviews^a^	
Single administration report (all symptoms, Fig. 1)	29 (100.0)
Specific symptom score in single administration report	29 (100.0)
Symptom over time, multiple administration report (Fig. 2)	29 (100.0)
Specific day in symptom over time report	29 (100.0)
Experience with SSPedi Completion	
Completed SSPedi more than once per day	2 (6.9)
Daily SSPedi completion frequency	
Too much	0
Too little	0
About right	29 (100.0)
Likelihood of daily completion	
1 – not likely at all	0
2	0
3	5 (17.2)
4	16 (55.2)
5 – very likely	8 (27.6)
Ease of completing SSPedi	
Very hard	0
Hard	0
Neither hard nor easy	1 (3.4)
Easy	8 (27.6)
Very easy	20 (69.0)

*Abbreviations*: *SSPedi* Symptom Screening in Pediatrics tool, *SPARK* Supportive care Prioritization, Assessment and Recommendations for Kids

^a^As assessed by two research associates rated on a 4-point Likert scale ranging from 1 = completely incorrect to 4 = completely correct, where correct was defined as a score of 3 or 4

Table 3 describes the facilitators and barriers to daily SSPedi completion. The most common barrier was symptom severity while the most common facilitator was the alarm feature. In terms of suggestions for modifications, only two were made. One suggested change was to make the default view for longitudinal reports show the 7 days prior to the last generated report while the second change was to correct an identified error in the programming.

**Table 3:** Barriers and facilitators to SPARK completion (*N* = 29)

Outcomes	Total n (%)
Barriers^a^	
No barriers	16 (55.2)
Symptom severity	9 (31.0)
Medical procedures	3 (10.3)
Website functionality (need to sign-in each day and identification of a programming error)	2 (6.9)
Facilitators^a^	
Alarm	18 (62.1)
Research associate visit	5 (17.2)
Family member	3 (10.3)
General interest	2 (6.9)

^a^Some participants provided more than one answer

## Discussion

We found that inpatient and ambulatory children with cancer and pediatric HSCT recipients were able to complete longitudinal, daily symptom screening over 5 days. Patients found the website easy to use, understood the symptom reports and were able to use the password and reminder system. This demonstration of feasibility is essential and allows progression toward a multi-center randomized trial to evaluate the efficacy of daily symptom screening with symptom feedback to healthcare professionals. This work is important as it provides a step-wise approach toward the creation of a system to promote symptom screening in a medically ill population, namely children receiving cancer treatments. Although it may have been possible to move directly to a randomized trial of longitudinal symptom screening, we believed that careful consideration of items such as password creation and reminders were important to ensure a future trial would be successful and ultimately to support clinical implementation.

To encourage daily symptom screening, we needed to create a feasible reminder system for this population. Studies in adult oncology evaluating electronic self-report symptom screening utilized a weekly email reminder or telephone call to encourage routine symptom reporting [10, 11]. Within the pediatric population, we were not confident that all patients would have an email or personal phone, and would access these systems daily while at the hospital. While the iPad reminder was successful, other approaches will likely be required for ambulatory patients with infrequent hospital visits and in this setting, a combination of tablet reminders and text messages for those with a mobile device may be optimal.

The ideal frequency of symptom screening is not known. Given that symptoms can change rapidly in these patients [12], we began with daily symptom screening but specifically solicited the opinions of our intended target population. While we asked for a minimum of daily symptom reporting, SPARK allows the patient to report symptoms multiple times in a given day and it was interesting to observe that two children chose to report their symptoms more than once per day in our study. While all enrolled children felt that daily symptom screening was about right (not too much and not too little), this may not be applicable to hospitalizations that are longer than 5 days. Identifying the ideal frequency of symptom screening both for inpatients with a prolonged hospitalization and for outpatients is an important research gap.

We observed excellent compliance with symptom screening in our study. It is possible that our high compliance rates were related to electronic rather than paper reporting of symptoms. Although there are some studies that have shown lower compliance with longitudinal reporting of paper-based patient-reported outcomes in pediatric cancer [13], given the different contexts, duration of reporting and specific supports to facilitate compliance, the merits of electronic versus paper reporting are difficult to compare directly.

A strength of this study was the step-wise approach toward developing a feasible approach to symptom screening for children receiving cancer treatments that considered many aspects important to implementation. The use of cognitive interviewing to evaluate utilization of SPARK is another strength. However the study is limited by its small sample size and conduct at a single center with only English-speaking children.

## Conclusions

Daily completion of symptom screening using SPARK over 5 days was feasible in children aged 8 to 18 years with cancer and pediatric HSCT recipients. SPARK is now appropriate for use in randomized trials to evaluate the efficacy of symptom screening and symptom feedback to healthcare professionals.

## Abbreviations

HSCT: Hematopoietic stem cell transplantation
SPARK: Supportive care Prioritization, Assessment and Recommendations for Kids
SSPedi: Symptom Screening in Pediatrics Tool
